# Planetary Gear Fault Diagnosis via Feature Image Extraction Based on Multi Central Frequencies and Vibration Signal Frequency Spectrum

**DOI:** 10.3390/s18061735

**Published:** 2018-05-28

**Authors:** Yong Li, Gang Cheng, Yusong Pang, Moshen Kuai

**Affiliations:** 1 School of Mechatronic Engineering, China University of Mining and Technology, Xuzhou 221116, China; liyong2015@cumt.edu.cn (Y.L.); kuaimoshen2016@cumt.edu.cn (M.K.); 2 Faculty Mechanical, Maritime and Materials Engineering, Delft University of Technology, Delft 2628, The Netherlands; Y.Pang@tudelft.nl

**Keywords:** planetary gear, fault diagnosis, VMD, center frequency, feature image, CNN

## Abstract

Poor working environment leads to frequent failures of planetary gear trains. However, complex structure and variable transmission make the vibration signal strongly non-linear and non-stationary, which brings big problems to fault diagnosis. A method of planetary gear fault diagnosis via feature image extraction based on multi central frequencies and vibration signal frequency spectrum is proposed. The original vibration signal is decomposed by variational mode decomposition (VMD), and four components with narrow bands and independent central frequencies are decomposed. In order to retain the feature spectrum of the original vibration signal as far as possible, the corresponding feature bands are intercepted from the frequency spectrum of original vibration signal based on the central frequency of each component. Then, the feature images of fault signals are constructed as the inputs of the convolution neural network (CNN), and the parameters of the neural network are optimized by sample training. Finally, the optimized CNN is used to identify fault signals. The overall fault recognition rate is up to 98.75%. Compared with the feature bands extracted directly from the component spectrums, the extraction method of the feature bands proposed in this paper needs fewer iterations under the same network structure. The method of planetary gear fault diagnosis proposed in this paper is effective.

## 1. Introduction

As a classic gear transmission mode, the planetary gear train is widely used in the transmission system of engineering machinery, aerospace and ship vehicles for its advantages of compact structure, large transmission ratio and strong bearing capacity. Poor conditions greatly increase the failure probability of planetary gear [1]. The transmission system is the core part of the power system. Its health condition seriously affects power transmission efficiency and the utilization ratio of the equipment. Moreover, if gear with minor faults cannot be detected and replaced in time, the degree of failure will be further aggravated. When a serious fault such as broken teeth occurs, it is very likely to cause serious accidents [2]. Therefore, detecting the fault mode of the gear in time and taking corresponding measures according to the failure condition are significant for safe and efficient operation of equipment.

Fault diagnosis can be roughly divided into two directions: fault pattern recognition and fault location. Compared with fault pattern recognition, different location faults have different fault frequencies, which make it a little easier. Fault feature frequency can be extracted based on signal de-noising and signal decomposition technology [3]. However, because different types of fault gear have the same fault location, the fault feature frequencies are the same. The method of fault location cannot be used to realize the fault diagnosis [4]. In order to solve this problem, Donatella proposed a fault gear diagnosis method based on the analysis of frequency sidebands. By analyzing the sideband features of the fault signals, the accurate identification of different degree of damage faults is realized [5]. It can be seen that the sideband, as a product modulated by different signals, can better respond to different types of gear failure modes. However, the structure of planetary gear train is complex, and the transmission path is changeable [6]. Strong non-linear and non-stationary features greatly increase the difficulty of extracting the sideband features [7]. Recursive adaptive decomposition methods such as empirical mode decomposition (EMD) and local mean decomposition (LMD) are widely used to deal with such signals [8,9]. Although these decomposition methods can better decompose simple signals, the phenomenon of modal aliasing begins to increase when dealing with complex signals. It is difficult to obtain components with narrow sideband [10]. Konstantin Dragomiretskiy proposed variational mode decomposition (VMD) in 2013, which is a new non-recursive decomposition algorithm for non-stationary signal processing [11]. The method is based on classical Wiener filtering, Hilbert transform and frequency mixing technology. The purpose is to find the center frequency band and the minimum bandwidth. It can adaptively realize frequency domain division and effective separation of components [12]. Compared with EMD, LMD and other improved algorithms, modal aliasing and endpoint effect are effectively solved. It also has strong robustness to noise and various applications such as signal de-noising and signal decomposition [13]. Based on this method, Feng et al. proposed a planetary gear fault diagnosis algorithm-based VMD and amplitude frequency joint demodulation. A representative fault frequency band is effectively found by VMD, which provides a stationary component signal for the amplitude frequency joint demodulation and greatly improves the accuracy of fault diagnosis [14].

As a signal feature, the sidebands can well reflect the differences between different fault signals. VMD can intercept the feature frequency band from the original vibration signal effectively [15]. However, in practical applications, it is found that in the modulation process of modal component and central frequency, the magnitudes of the frequency spectrum are changed. The center part of the frequency spectrum is amplified, and the amplitudes of the sideband gradually weakens. It makes the large number of original details cannot be retained in the sideband. In order to solve this problem, this paper proposes a feature frequency interception algorithm based on the central frequencies of VMD components and the spectrum of the original vibration signal. First, the representative feature components and their center frequencies in the signal are extracted by decomposing the original vibration signal with VMD; then taking the central frequency of each component as the center, the feature frequency bands are intercepted from the frequency spectrum of the original vibration signal.

Extracting feature values based on basic data is the key step of fault diagnosis. A reasonable selection of sensitive features and high-quality classification neural network can achieve a better fault recognition result [16]. Chen et al. proposed a method for diagnosing faults in planetary gear based on fuzzy entropy of Local mean decomposition (LMD) and Adaptive neuro-fuzzy inference system (ANFIS), which realized the classification of different types of fault patterns. The comprehensive recognition rate reached 88.8% [17]. However, there are certain differences in the structure of different transmission systems, and the selection of sensitive fault features is often more pertinent. The overall recognition algorithm is lack of universality, which greatly reduces the practical use value of the algorithm [18]. Studies have shown that the multilayer mechanism of mammalian cerebral cortex is the main reason for capturing information and rules and identifying objects. The brain processes information and identifies objects after many times of aggregation and decomposition [19]. Based on this principle, HINTON proposed deep learning theory in 2006. This method perfectly integrates feature extraction, data reduction and pattern recognition. Pre-processed data can be directly used as a sample to train deep network [20]. Compared with traditional classification and recognition models, it has many advantages such as universality, simple structure and high recognition rate [21]. As a special deep neural network model, convolutional neural network (CNN) has been widely applied in the field of image recognition. Its network structure of non-full join and weight sharing makes it more similar to biological neural network, and reduces the complexity of network model [22]. Cui et al. proposed a tire defect classification method based on multi comparison CNN, which improved the accuracy of tire defect classification under different illumination [23]. In this paper, this method is introduced to the fault pattern recognition of the planetary gears. First, using the feature frequency extraction method proposed in this paper, the feature spectrum of the vibration signal is constructed as CNN input. Then training samples are used to optimize the network parameters. Finally, the final fault pattern recognition is realized.

## 2. Model Building

In this paper, a method of planetary gear fault diagnosis via feature image extraction based on multi central frequencies and original vibration signal frequency spectrum is proposed. The method is roughly divided into four steps. Firstly, VMD is used to acquire modal components with prominent features. Secondly, based on the central frequency of modal component, the feature frequency band is extracted from the original vibration signal spectrum. Then, the feature frequency band is split up and reorganized to get the signal feature spectrum image. Finally, the image samples are used to train CNN and realize fault diagnosis.

### 2.1. Variational Mode Decomposition

As a non-recursive adaptive decomposition method, VMD combines classical Wiener filtering, Hilbert transform and frequency mixing technology [24]. Suppose that each mode has limited bandwidth with center frequency, the central frequency $\omega_{k}$ and modal function $u_{k}$ of the decomposition process are continuously updated in the decomposition process to seek the *K* modal functions with the least sum of bandwidth, k=1, 2, 3, …,K. The algorithm flow is as follows:

Step 1: Initialize modal component $u_{k}$, center frequency $\omega_{k}$, tolerance of convergence criterion *e*, number of iterations *N*, quantity of components *K* and moderate bandwidth constraint α.

Step 2: Each modal function $u_{k}$ is carried out by Hilbert transformation and mixed with the estimated center frequency of each modal component. The spectrum of each mode is modulated to the corresponding fundamental frequency band.

(1) $$[(\delta (t)+\frac{j}{\pi t})u_{k}(t)]e^{-j\omega_{k}t}$$

where *t* is time and δ(t) is an impact function.

Step 3: Establish a constrained variational model. The two-penalty factor α and the Lagrange multiplication operator λ(t) are introduced to construct a Lagrange expression, it is as follows:

(2) $$L(\{u_{k}(t)\},\{\omega_{k}\},\lambda (t))=\alpha \sum_{k=1}^{K}{\left\|\frac{\partial \left[\delta (t)+\frac{j}{\pi t}\right]u_{k}(t)}{\partial t}e^{-j\omega_{k}t}\right\|}_{2}^{2}+{\left\|f(t)-\sum_{k=1}^{K}u_{k}(t)\right\|}_{2}^{2}+\langle \lambda (t),f(t)-\sum_{k=1}^{K}u_{k}(t)\rangle$$

where the two-penalty factor α guarantees the reconstruction accuracy of the signal in the presence of Gauss noise, and the Lagrange operator λ(t) keeps the constraint condition strictly.

Step 4: The method of alternating direction multiplier algorithm is used to solve the above variational problems. The saddle point of the extended Lagrange expression is obtained by altering the update parameters $u_{k}(t)$, $\omega_{k}$ and λ(t). The update formula is as follows.

(3) $$\;\overset{\wedge }{u_{k}^{n+1}}(\omega )=\frac{\overset{\wedge }{f}-\sum_{i\neq k}\overset{\wedge }{u_{i}}(\omega )+\frac{\overset{\wedge }{\lambda }(\omega )}{2}}{1+2\alpha {\left(\omega -\omega_{k}\right)}^{2}}$$

(4) $$\omega_{k}^{n+1}=\frac{{\int_{0}^{\infty }\omega |\overset{\wedge }{u_{k}}(\omega )|}^{2}d_{\omega }}{{\int_{0}^{\infty }\left|\overset{\wedge }{u_{k}}(\omega )\right|}^{2}d_{\omega }},k\in \left\{1,2,\ldots ,K\right\}$$

(5) $$\overset{\wedge }{\lambda^{n+1}}(\omega )=\overset{\wedge }{\lambda^{n}}(\omega )+\tau (\overset{\wedge }{f}(\omega )-\sum_{k}\overset{\wedge }{u_{k}^{n+1}}(\omega ))$$

Step 5: When meeting the iteration termination condition (6) or (7), the update of parameters is stopped, and the VMD is completed.

(6) $$\sum_{k=1}^{K}\frac{\overset{\wedge }{u_{k}^{n+1}}(\omega )-\overset{\wedge }{u_{k}^{n}}(\omega )}{\left||\overset{\wedge }{u_{k}^{n}}|\right|}<\varepsilon$$

(7) n≥N

### 2.2. Feature Image Construction

The edge band can well reflect the difference between different fault signals. VMD can be used to intercept the most feature frequency band in the original vibration signal. However, it is found that in the modulation process of modal component and central frequency, the magnitude of the original vibration signal spectrum is changed, which results in the large number of original details that cannot be retained in the sideband. In order to solve this problem, this paper proposes an interception algorithm of feature frequency band based on multi central frequencies and vibration signal frequency spectrum. The algorithm is divided into two steps:

Step 1: By decomposing the original vibration signal with VMD, the representative feature components are extracted and the central frequency of each component is obtained.

Step 2: The center frequency is set to the center of the feature frequency band, and the feature frequency band is cut from the original spectrum.

Step 3: The components are split into several segments, and then each segment is sorted by columns. Finally, a nearly square feature spectrum image is obtained.

### 2.3. Convolutional Neural Network

**Figure 1:**
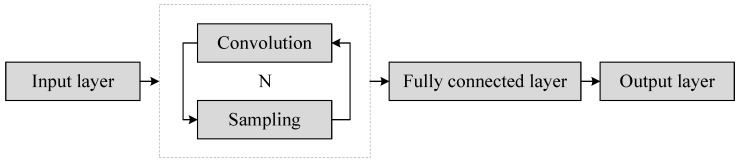
The simplified structure of deep neural network based on convolutional neural networks (CNNs).

CNN can effectively establish mapping relationship between feature spectrum and fault types. The network is usually composed of 5 functional networks: input layer, convolution layer, pool layer, full connection layer and output layer. The input layer is used to implement the sample input, the convolution layer and the sampling layer are used to realize the adaptive feature extraction and dimensionality reduction, the full connection layer and the output layer are used to realize pattern recognition [25]. The simplified structure of deep neural network based on CNN is shown in Figure 1.

The whole training process is divided into 6 steps:

Step 1: Select appropriate group number of convolution-pooling *L*, convolution kernel size *c* and full connection layer size *z* based on input samples and output categories.

Step 2: Set learning rate η, number of iterations *T* and batch size *S*. Initialize network weights $w_{l}$ and biases $b_{l}$.

Step 3: Use convolution kernel to do convolution operation and increase the bias to the result. Sigmoid function is used to select output features:

(8) $$x_{j}^{l}=f(\sum_{i\in M_{j}}x_{i}^{l-1}\times c_{i,j}^{l}+b_{j}^{l})$$

where $M_{j}$ represents the input feature atlas, *l* represents the number of layers, and *f* is the excitation function.

Step 4: The maximum value in each m×m field is taken from convolution feature graph. A new feature map with a *m* fold reduction is produced by adding bias and excitation function.

(9) $$x_{j}^{l}=f(\max(down(x_{j}^{l-1}))+b_{j}^{l})$$

where *j* is the sequence number of the feature map, down (*) is the down sampling function, and max (*) indicates the maximum pooling method.

Step 5: Repeat steps 4 and 5 according to network structure until *l* reaches the level of training *N*. The mapping relationship between the fully connected layer and the input layer is established to complete the whole network construction.

Step 6: Unfold all the features and select the classifier. The mapping relationship between the fully connected layer and the input layer is established to complete the whole network construction. Samples are used to train neural network and optimize network parameters. When *t* reaches to the number of iterations *T*, the network training is completed.

## 3. Experimental Equipment

**Figure 2:**
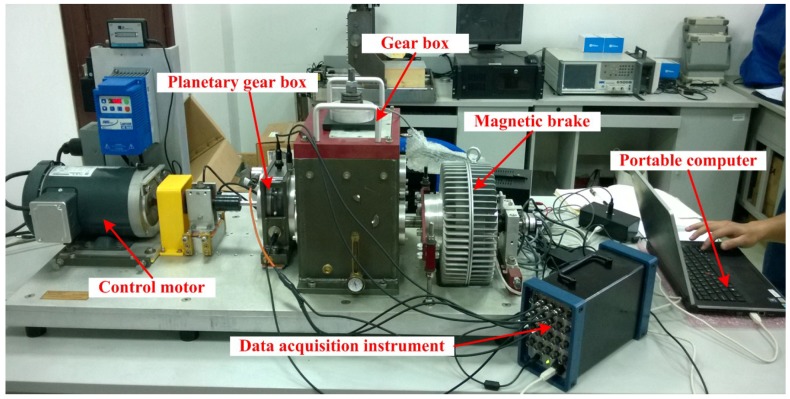
Drivetrain Dynamics Simulator (DDS) comprehensive test bed for fault diagnosis.

**Figure 3:**
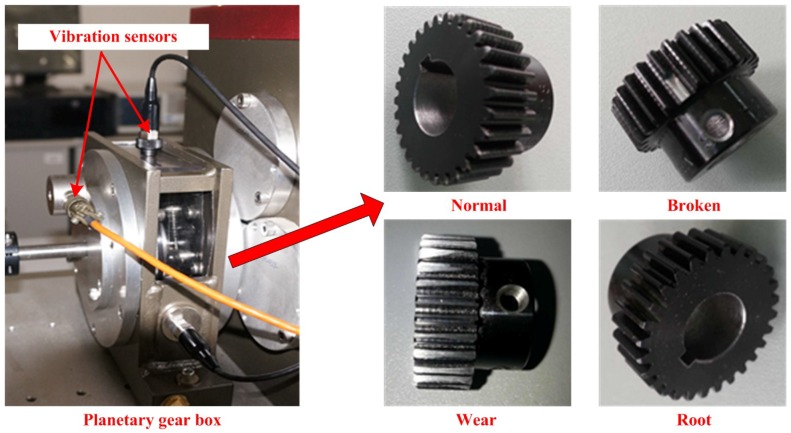
Planetary gear boxes and different fault sun gears.

Planetary gear fault experiments were simulated in the Drivetrain Dynamics Simulator (DDS) mechanical fault comprehensive simulation produced by Spectra Quest Company. The experimental system is composed of a two-stage planetary gearbox, a programmable control motor, a fixed-axis gearbox, a programmable magnetic brake component, a data acquisition system and a portable computer. The simulation bench is shown in Figure 2. All types of gear vibration signals are measured by acceleration sensors, the layout of which is shown in Figure 3.

The output frequency of the motor controlled is set as 45 Hz. In addition, the load controlled by a programmable magnetic brake component is set as 13.5 nm, In the experiments, the sampling frequency is set as 12,800 Hz. The original vibration signal is mainly composed of motor rotation frequency, gear meshing frequency, planet carrier rotation frequency and fault feature frequency. The frequency of each component is shown in Table 1.

## 4. Experimental Analysis

**Figure 4:**
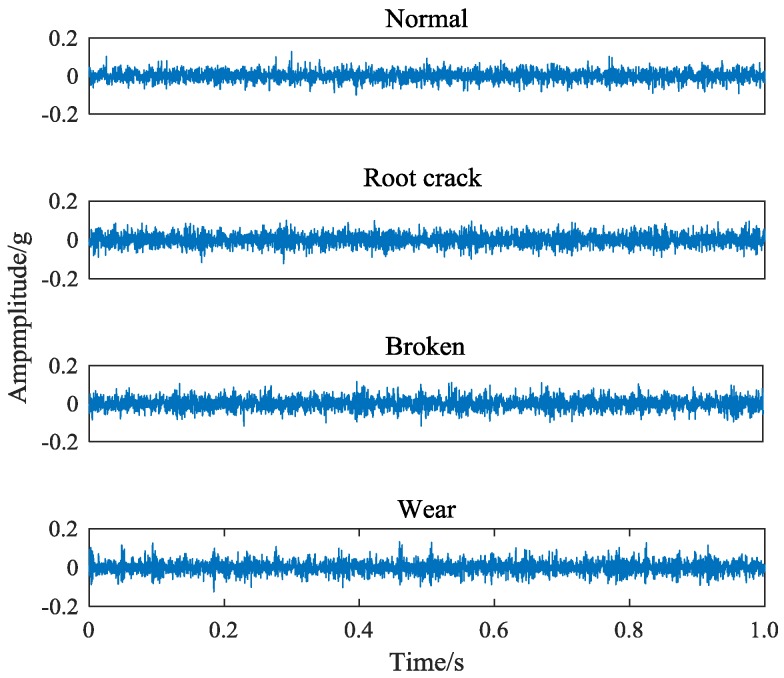
Time-domain signals of four different gear states.

Taking the sun wheel as the research object, four sets of data under normal, wear, broken and crack state were collected. The vibration signals collected from different types of failures are shown in Figure 4.

**Figure 5:**
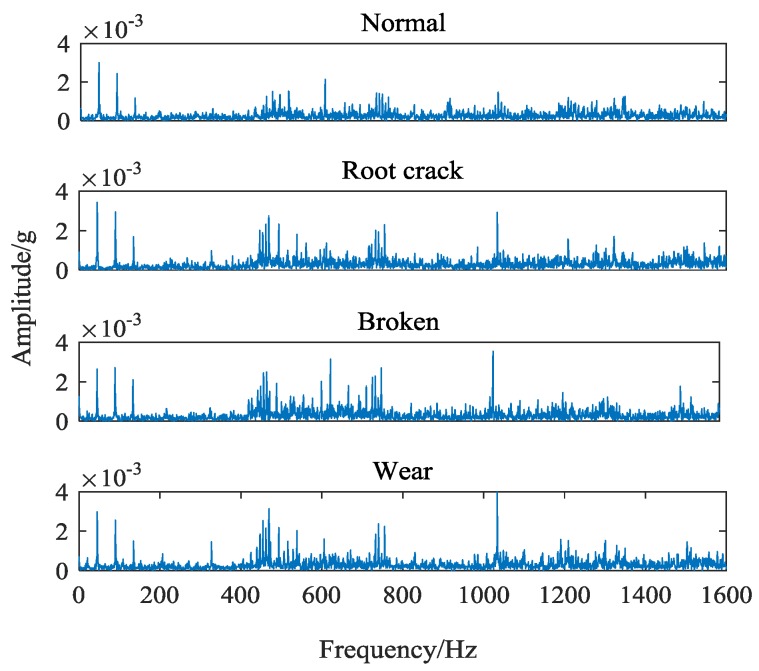
Frequency-domain signals of four different gear states.

It can be seen that because of the consistent fault location and the high similarity of the components of the signal, the obvious differences cannot be seen from the time domain signal of different faults. While the different types of fault in the meshing condition, the subtle fault difference will affect the frequency and band of the main center of the signal, which makes a certain difference in the spectrum diagram of the vibration signal. Frequency-domain signals of four different gear states are shown in Figure 5. This paper takes the gear vibration signal of the tooth root crack status as an example and starts with the difference of spectrum.

**Figure 6:**
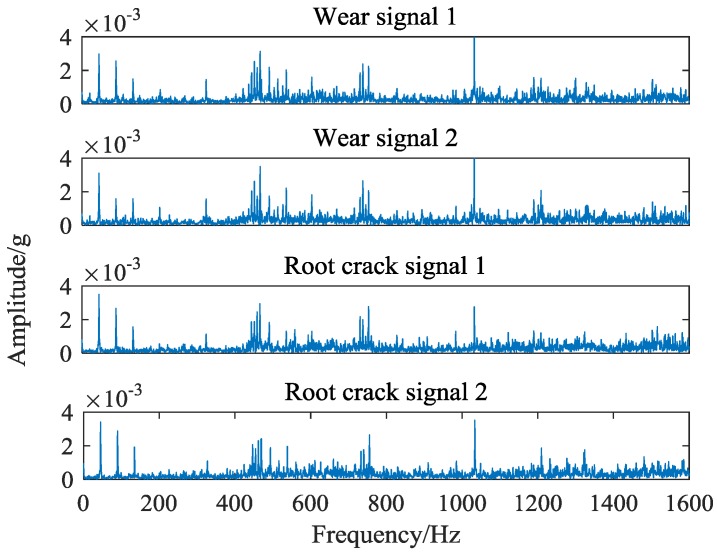
Highly similar vibration signals: wear and root crack.

It can be seen from Figure 5, the magnitude and distribution of the spectrum amplitude between different types of fault signals are different. However, this difference is relatively small in proportion to the whole spectrum, and is easily affected by the essential difference of signals collected in different periods. Moreover, the spectrum similarity between root cracks and wear faults is high, which is shown in Figure 6.

It is not conducive to judging the fault types directly through spectrum. Therefore, it is necessary to carry out further research from the signal spectrum to achieve the final fault diagnosis.

**Figure 7:**
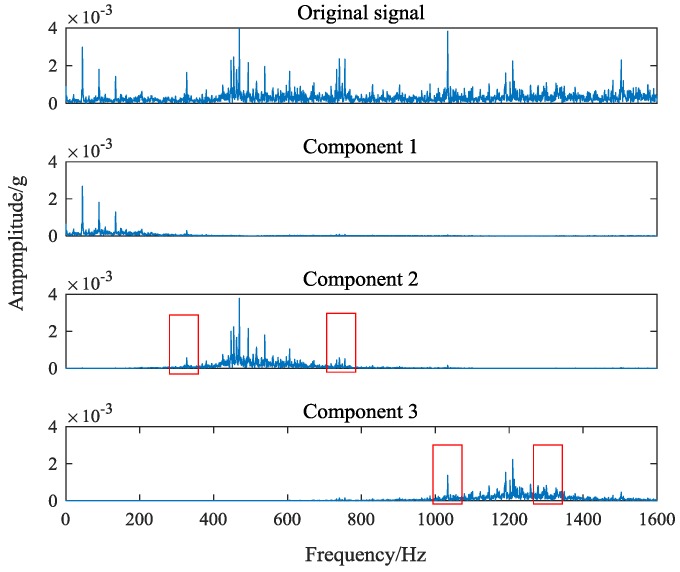
Component spectrum obtained by variational mode decomposition (VMD) (k=3, α = 500).

Signal decomposition plays an important role in signal processing. Better decomposition can effectively extract representative feature components from the original vibration signal and amplify the features. As a non-recursive adaptive decomposition algorithm, VMD avoids the modal aliasing and endpoint effects in recursive EMD and LMD decomposition. Based on the classical Wiener filtering, Hilbert transform and frequency mixing technique, the frequency center and bandwidth of each decomposition component are determined by iterative searching the optimal solution of the variational model. The frequency domain dissection and the effective separation of the components can be realized adaptively. However, it is found that in the modulation process of modal component and central frequency, the magnitude of the vibration signal frequency spectrum is changed, which results in the large number of original details that cannot be retained in the sideband. Figure 7 shows the spectrum of signal components obtained by VMD (k=3, α = 500).

Figure 7 shows that VMD effectively selects feature components in signals. However, the amplitude of the signal edge frequency is severely weakened in the component 2 and the component 3. The change of the amplitude makes the feature frequency of the original vibration signal cannot be extracted. This is caused by VMD in order to minimize the sum of bandwidth when modulating modal components and central spectrum. In order to solve this problem, this paper proposes an interception algorithm of feature frequency band based on multi central frequencies and frequency spectrum of original vibration signal. First, the representative feature components and their center frequencies in the signal are extracted by decomposing the original vibration signal with VMD; then taking the central frequency of each component as the center, the feature frequency bands are intercepted from the original vibration signal spectrum.

The core parameters of VMD are component number k and sideband constraint α. *K* determines the number of components decomposed by signal and α determines its spectrum bandwidth. When *k* is large, there will be over decomposition phenomena, such as noise components or overlapped components. When *k* is small, local features will be ignored and cannot appear in separate components. By observing the original vibration signal frequency spectrum of four kind gear states in Figure 4, it can be seen that there are probably 4–5 main peaks in each signal spectrum. Because the purpose of feature extraction in this paper is to extract the center frequency and sideband features, it only needs to get the most feature signal components. So it is unnecessary to get every component of the signal completely. Furthermore, the difference of signals can affect the selection of center frequency. The smaller *K* value can effectively avoid the appearance of noise components and increase the difference of feature spectrum. Therefore, the number of components is set as 4. In order to avoid the occurrence of modal aliasing, the sideband bandwidth of each component should be controlled as much as possible. When *k* = 4, the decomposition effects of the signal under different α values are tested. The distribution of the central frequency of each component and the size of the band bandwidth are obtained, as shown in Table 2.

**Figure 8:**
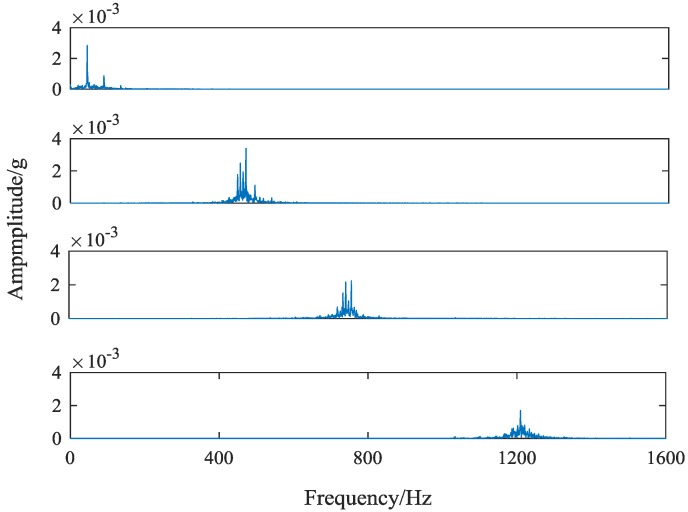
The feature frequency bands extraction based on component frequency spectrum.

It can be seen from Table 2 that the center frequencies fluctuate within a certain range under the influence of the bandwidth constraint parameter α. However, the approximate distances between each other remain unchanged. The closest one is the central frequency of the second component and the central frequency of the third component, with a distance of about 240 Hz. To effectively avoid modal aliasing, the sideband bandwidth of the spectrum should be controlled within 240 Hz. With the increase of α, sideband bandwidths are shrinking. When α = 10,000, sideband bandwidths are controlled within acceptable limits. Therefore, the value of α is set as 10,000. Figure 8 shows the spectrum of every component after VMD decomposition.

**Figure 9:**
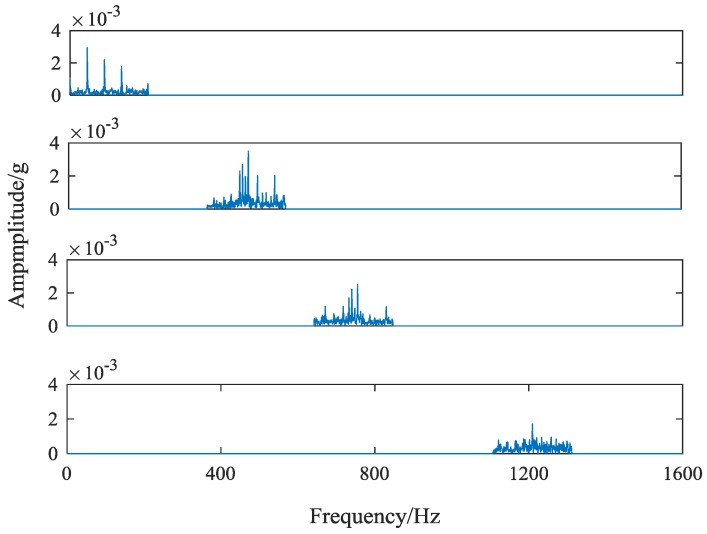
The feature frequency bands extraction based on original signal frequency spectrum.

Compared with the original vibration signal frequency spectrum, it can be found that the amplitude reduction of spectrum edge is serious. Next, the method of this paper is used to intercept the feature frequency spectrums. After Fourier transform, each component signal contains 1600 frequencies, corresponding to 2048 sampling points. The sideband bandwidth of each component is around 200 Hz, and the number of sampling points corresponding to the 200 Hz bandwidth is 256. The interception criteria of the feature band are as follows: taking the sampling points corresponding to the central frequency of each component as the center, 127 sampling points are fetch forward and 128 sampling points are taken backwards. When the sampling point corresponding to the center frequency is less than 128, the first 256 sampling points are taken. The frequency spectrum of each component obtained by this method is shown in Figure 9.

By comparing the two graphs of Figure 7 and Figure 8, it can be seen that the extraction method of feature frequency band based on the center frequency of each component and the spectrum of the original vibration signal not only effectively extracts the most prominent central frequency of various fault gear vibration signals, but also effectively preserves the original feature frequency bands near the center frequency. The feature frequency bands extracted well reflect the feature difference of different gear states, providing high-quality basic data for subsequent fault classification.

Feature extraction is the core component of fault diagnosis technology. The quality of feature extraction often determines the final fault recognition effect. However, traditional features such as energy features, entropy features and so on, depend on their data strongly and have poor universality. The same method applied to different working conditions is often not ideal. The deep neural network based on multi-layer CNNS has good generality and high recognition rate. This method is widely used in the field of image recognition with its ultra-high recognition rate. LeNet-5 is a classical CNN model which is the first CNN successfully applied to handwritten numeral recognition. On the Modified National Institute of Standards and Technology (MNIST) data set, the recognition rate of LeNet-5 model can reach about 99.2%. The LeNet-5 model has 7 layers, where the convolution kernel size is 5×5, the pool level filter is 2×2, and the two-level full connection layer number is 120 and 84. In this paper, it is introduced into the fault diagnosis of planetary gear, and on this basis, its structure is modified.

**Figure 10:**
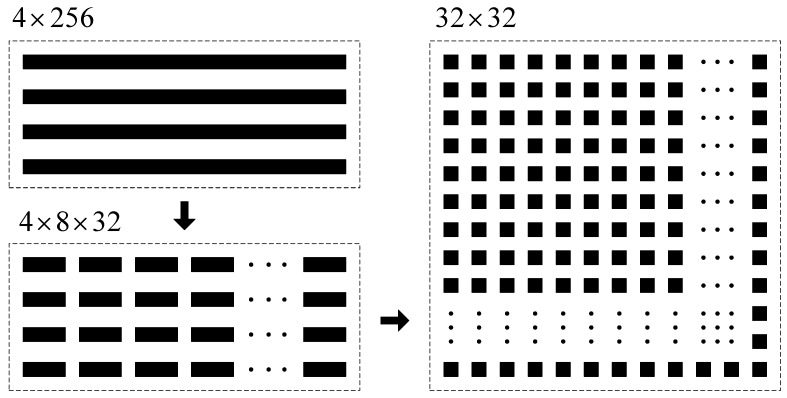
Construction process of the feature image.

The original vibration signal is processed by VMD, and the feature frequency band with a length of 256 is intercepted based on the central frequency of each component. The feature matrix of dimension 4×256 can be obtained. In this paper, the feature matrix of 4×256 is split by row, and every row is split into 8×32 matrix. Then, the 4 matrices are sorted by columns, and the feature matrix of 32×32 can be obtained. The construction process of the feature image is shown in Figure 10.

**Figure 11:**
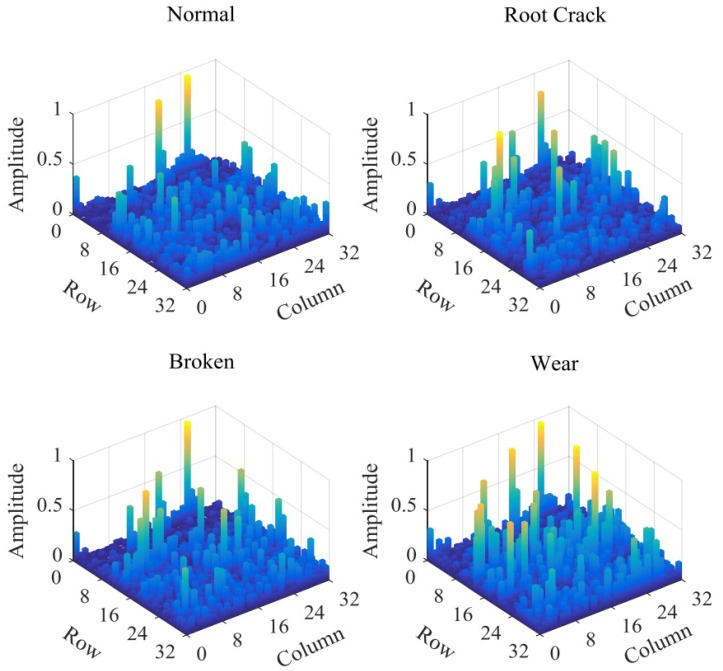
Feature matrix of four gear states.

In order to facilitate network operation, the amplitudes of feature matrix are normalized. The normalization of the characteristic matrix provides a great help for the later convolution network training. The same amplitude range reduces the fluctuation of weights, bias and other parameters in the network, which reduces the amount of computation and improves the quality of the network effectively. The feature matrix of the four faults is displayed in the form of three-dimensional bar graph, as shown in Figure 11.

As can be seen from Figure 11, by three steps of VMD, Fourier transform and characteristic matrix construction, different types of original vibration signals are displayed in the form of images with size of 32×32. The distribution of high amplitude feature points has obvious differences. The feature matrix amounts to 1024 data. Compared with the original frequency signal with 2048 sampling points in Figure 5, the number of features drops to half the level, which greatly reduces the amount of computation. In addition, the size of the matrix satisfies the classical neural network LeNet-5 model, which provides a great help for obtaining good recognition results.

**Figure 12:**
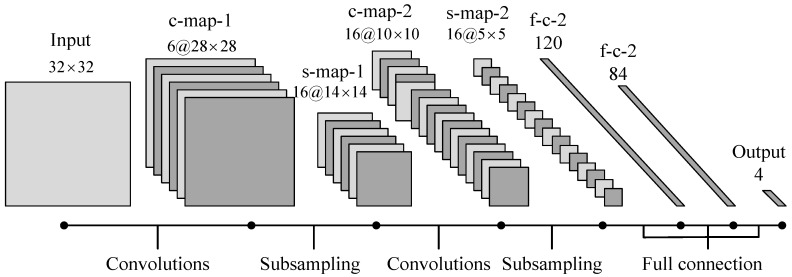
Architecture of the LeNet-5 model.

The output is the four state of gear failure, the number of neurons in output layer is 4. The other parameters remain unchanged. Figure 12 shows the modified architecture of the LeNet-5 model.

**Figure 13:**
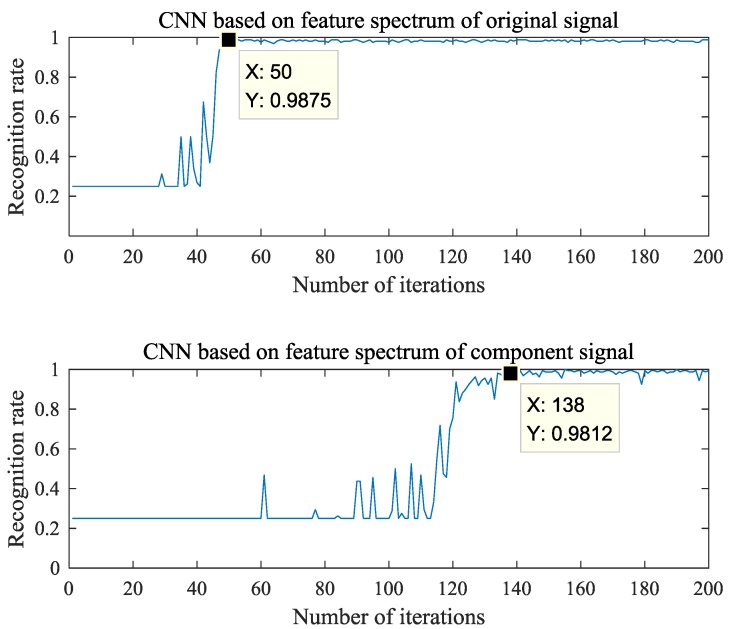
Recognition rate varies with the number of iterations.

In this experiment, four groups of vibration signal samples were collected from normal, broken, wear and crack states. Each group has 120 segments of signals, and the four groups have 480 segments of signals. 320 segments are used for training deep neural network, and the 160 is for testing. At the same time, two kinds of feature band extraction algorithms were tested by CNN. One is to intercept the feature frequency band from the frequency spectrum of each component based on the central frequency of each component. The other is to intercept the feature frequency band from the frequency spectrum of original vibration signal based on the central frequency of each component. The application effects of two methods are shown in Figure 13.

As can be seen from Figure 12, the interception method of feature frequency spectrums based on the center frequency of each component and the frequency spectrum of original vibration signal can obtain a higher stability recognition rate of 98.75% with less iteration times, 50 times. The feature frequency interception method based on the center frequencies of each component and the component signal spectrums requires 138 iterations to obtain a more stable recognition effect, and the recognition rate is 98.12%. Therefore, the method proposed in this paper can effectively extract the feature frequency spectrums of the signal. It can further highlight the difference features of the signal and provide excellent basic data for the realization of signal recognition.

## 5. Conclusions

This paper proposes a method of planetary gear fault diagnosis via feature image extraction based on multi central frequencies and vibration signal frequency spectrum. Considering the high similarity of the vibration signals of different gear types, this paper starts with the study of the sideband of the signal. Firstly, in view of strong non-linear non-stationary features of signals caused by the complex components and the variable transmission path, VMD is used to decompose original vibration signal and four representative feature components and center frequencies are extracted. Secondly, in order to overcome the problem of edge spectrum weakening in component spectrums, an interception method of feature spectrum based on component center frequencies and frequency spectrum of original vibration signal is proposed in this paper. Taking the central frequency of each component as benchmark, the corresponding frequency bands are cut from the frequency spectrum of original vibration signal, which effectively preserves the features of the original sidebands near the center frequencies. In addition, the four most featured frequency bands with a length of 256 are obtained from the original frequency spectrum. Then, the intercepted feature bands are used to construct a feature matrix with a size of 32×32 and serve as the input data of CNN. Finally, the network is optimized through sample training, and the recognition of fault signal types is realized. The overall recognition rate can reach 98.75%. The proposed method is further illustrated to be effective in fault diagnosis of planetary gears.

## Figures and Tables

**Table 1:** Components of vibration signals and their corresponding frequencies.

Name	Frequency/Hz
Motor rotation frequency	45
Meshing frequency of the first stage planetary gear	750
The rotational frequency of the first stage planetary frame	7.5
Meshing frequency of the second stage planetary gear	164
Fault frequency of the second stage solar wheel	23.4

**Table 2:** Center frequencies and frequency bandwidth under different value α.

Value α	Center Frequencies/Hz				Bandwidth/Hz
	Component 1	Component 2	Component 3	Component 4	
2000	72.6	486.4	726.4	1289.3	400
4000	71.0	484.2	723.5	1281.3	350
6000	74.2	483.2	723.9	1298.6	310
8000	55.7	481.9	724.8	1196.5	260
10,000	50.9	481.0	725.1	1191.0	220
